# Full-Length Fibronectin Drives Fibroblast Accumulation at the Surface of Collagen Microtissues during Cell-Induced Tissue Morphogenesis

**DOI:** 10.1371/journal.pone.0160369

**Published:** 2016-08-26

**Authors:** Jasper Foolen, Jau-Ye Shiu, Maria Mitsi, Yang Zhang, Christopher S. Chen, Viola Vogel

**Affiliations:** 1 Laboratory of Applied Mechanobiology, Department of Health Sciences and Technology, ETH Zurich, Vladimir-Prelog-Weg 4, Zurich, Switzerland; 2 Department of Biomedical Engineering, Boston University, Boston, Massachusetts, United States of America; 3 Wyss Institute for Biologically Inspired Engineering, Harvard University, Boston, Massachusetts, United States of America; University of California Berkeley, UNITED STATES

## Abstract

Generating and maintaining gradients of cell density and extracellular matrix (ECM) components is a prerequisite for the development of functionality of healthy tissue. Therefore, gaining insights into the drivers of spatial organization of cells and the role of ECM during tissue morphogenesis is vital. In a 3D model system of tissue morphogenesis, a fibronectin-FRET sensor recently revealed the existence of two separate fibronectin populations with different conformations in microtissues, i.e. ‘compact and adsorbed to collagen’ versus ‘extended and fibrillar’ fibronectin that does not colocalize with the collagen scaffold. Here we asked how the presence of fibronectin might drive this cell-induced tissue morphogenesis, more specifically the formation of gradients in cell density and ECM composition. Microtissues were engineered in a high-throughput model system containing rectangular microarrays of 12 posts, which constrained fibroblast-populated collagen gels, remodeled by the contractile cells into trampoline-shaped microtissues. Fibronectin’s contribution during the tissue maturation process was assessed using fibronectin-knockout mouse embryonic fibroblasts (Fn^-/-^ MEFs) and floxed equivalents (Fn^f/f^ MEFs), in fibronectin-depleted growth medium with and without exogenously added plasma fibronectin (full-length, or various fragments). In the absence of full-length fibronectin, Fn^-/-^ MEFs remained homogenously distributed throughout the cell-contracted collagen gels. In contrast, in the presence of full-length fibronectin, both cell types produced shell-like tissues with a predominantly cell-free compacted collagen core and a peripheral surface layer rich in cells. Single cell assays then revealed that Fn^-/-^ MEFs applied lower total strain energy on nanopillar arrays coated with either fibronectin or vitronectin when compared to Fn^f/f^ MEFs, but that the presence of exogenously added plasma fibronectin rescued their contractility. While collagen decoration of single fibronectin fibers enhanced the non-persistent migration of both Fn^f/f^ and Fn^-/-^ MEFs, the migration speed was increased for Fn^-/-^ MEFs on plasma fibronectin fibers compared to Fn^f/f^ MEFs. In contrast, the average speed was the same for all cells on collagen-coated Fn fibers. A Fn-FRET sensor revealed that fibronectin on average was more extended on the microtissue surface compared to fibronectin in the core. Gradients of collagen-to-fibronectin ratios and of the fraction of collagen-adsorbed to stretched fibrillar fibronectin conformations might thereby provide critical cell migration cues. This study highlights a dominant role for fibronectin in tissue morphogenesis and the development of tissue heterogeneities.

## Introduction

Healthy connective tissue maintains a function-specific hierarchical organization of cells and ECM. Formation of tissue anisotropy is thereby driven by biochemical gradients and physical factors, including the need to bear the imposed loads while ascertaining *in vivo* tissue functionality and durability [1,2,3,4]. However in pathological conditions, external overloading or inflammation of fibrous tissue can cause a progressive increase in cell and matrix reorganization, as in atherosclerosis [5], ventricular overloading [6], cardiomyopathy [7] and tendon overload [8]. In addition, replicating and maintaining this native-like architecture of ECM and cells when engineering tissues *in vitro*, still remains a challenge. In tissue engineering, where homogeneous polymeric scaffolds are typically presented to cells, the density of cells and associated ECM are found to progressively increase at tissue surfaces [9,10,11,12,13], and in thin tissues this cannot solely be attributed to diffusion limitations of the supplied nutrients. For example, oxygen limitations only start at a tissue depth of 200μm [14]. Recently, the increase in fibroblast density towards tissue surfaces in reconstituted collagenous microtissues, approximately 40μm thick, was correlated with an increasingly anisotropic distribution of fibronectin (Fn) [10,13,15]. The emerging shell-like tissues contained a collagen-rich tissue core that was sparsely populated by cells, but progressively covered by fibronectin-rich surface layers containing high cell densities [10]. However, insight is still missing regarding the underlying mechanisms that drive tissue morphogenesis to generate such cell density gradients. In particular, it is unknown what the role of ECM is and how it affects spatial gradients of the associated processes, such as cell migration and force generation. In the current study we asked how fibronectin in the context of collagen gels impacts cell-induced tissue morphogenesis, specifically the depth-dependent distribution of cells and their ECM.

Fibronectin is a dimeric glycoprotein that consists of two almost identical monomers [16,17,18]. Cellular fibronectin, as produced by fibroblasts and many other cells, contains additional segments compared to plasma fibronectin, notably, the EDA and EDB domains [16,17,18,19,20]. Plasma fibronectin is produced solely by hepatocytes [16] and circulates in the blood plasma, as well as in other body fluids. Both plasma and cellular fibronectin interact with cells mostly via the classic integrins αvβ3 and α5β1, but plasma and cellular fibronectin show distinct downstream effects on cell signaling [19,20,21,22,23,24,25,26], and were thus shown to have distinct and independent functions during tissue repair [22]. For cellular fibronectin, the EDA domain contains binding sites for α4β1 and α9β1, while no binding sites were identified so far on the EDB domain [16]. While cells probe their environment, they pull, stretch and deflect the ECM fibers to which they are attached until homeostasis is reached, or until they start to migrate. Fibroblasts are unable to migrate into plasma clots when the clots are depleted of fibronectin [27]. A similar phenomenon is observed for sprouting microvessels from rat aorta explants that are embedded in rat tail collagen gels [28]. The length of these sprouting microvessels was observed to increase with increasing concentrations of plasma fibronectin added to the collagen gel [28]. The addition of fibronectin was also observed to promote migration and matrigel invasion by ovarian cancer cells, in a focal adhesion kinase (FAK)-dependent manner [29]. Since mechanical forces acting on ECM fibers result in conformational changes of fibronectin through stretching and partial unfolding of its domains which all carry specific binding sites [30,31,32,33], fibronectin can be regarded as a mechanotransducer [34]. Stretch-induced conformational changes of the protein exposes cryptic binding sites for fibronectin self-association [32,33,35], reduces the affinity to bacterial adhesins [36,37] and promotes the osteogenic differentiation of human mesenchymal stem cells [38]. Furthermore, the otherwise hidden synergy site, which enhances α5β1-integrin binding to fibronectin, gets exposed when cells pull on fibronectin anchored to rigid but not to soft polyacrylamide substrates, and the exposure of the synergy site correlates with fibronectin-mediated FAK activation in fibrocarcinoma cells [39].

Major synergies exist between fibronectin and other ECM components during tissue remodeling and morphogenesis [20]. While it was initially reported that cellular but not plasma fibronectin is required to induce collagen gel contraction [40], it was later shown that fibronectin-knockout mouse embryonic fibroblasts (Fn^-/-^ MEFs) are still able but limited in their ability to contract type I collagen gels prepared in fibronectin-depleted growth medium (only a ~30% reduction in gel volume was observed) [41], whereby gel contractions up to ~80% is observed with the addition of exogenous plasma fibronectin [41]. Also inhibiting the interaction between collagen and fibronectin through the addition of a fibronectin-to-collagen binding inhibitor (R1R2), reduces gel contraction by Fn^-/-^ MEFs in a dose-dependent manner [42] and severely affects tissue morphogenesis of 3D collagen-fibronectin tissue [43]. Additionally, contraction of a gel mixture of Fn^-/-^ MEFs, type I collagen and fibronectin was significantly reduced upon treatment of the gel with anti-β1 or -α5 integrin antibodies [41]. Fibronectin can thus promote cell-induced collagen gel contraction via the most dominant cell-fibronectin binding integrin, α5β1 [44]. Not surprisingly, fibronectin provides a template for type I and III collagen assembly in tissue repair and development [45,46].

There is a specific role for fibronectin in regulating tissue morphogenesis, as already well established in early development, wound healing and in cancer progression. Model systems using cell-contracted collagen gels gave further insights how fibronectin impacts cell-induced tissue morphogenesis and the resulting heterogeneous distribution of cells and ECM components. By using FRET-labeled fibronectin to sense the conformation of fibronectin in contracted collagen microtissues, Legant et al. [10] showed that two different populations of fibronectin exist, i.e. collagen-absorbed, more compact fibronectin, as well as cell-assembled fibrillar fibronectin that was stretched by cell-generated forces [10]. Interestingly, cells were increasingly located at tissue surfaces with progression of culture time, correlating with the highest densities of fibrillar fibronectin, but the reason why the cells accumulated on the surface of the contracted gels remained unclear.

Here, the contribution of fibronectin to the collagen gel microtissue morphogenesis was studied using fibronectin-knockout mouse embryonic fibroblasts (Fn^-/-^ MEFs) and floxed equivalents (Fn^f/f^ MEFs) [47] in combination with a FRET-fibronectin sensor that can directly bind to collagen, as unlabeled fibronectin does as well, and gets incorporate by fibroblasts into their ECM fibrils [30,31]. Since we found here that the presence of plasma fibronectin in the medium, but not of its fragments, has a major effect on tissue morphogenesis by Fn^-/-^ MEFs, cellular traction forces were measured using nanopillar arrays in a secondary series of experiments, while migration properties were determined by tracking migrating single cells on single manually pulled fibronectin fibers.

## Results

### Fibronectin is required for the accumulation of cells at the microtissue periphery

**Fig 1:**
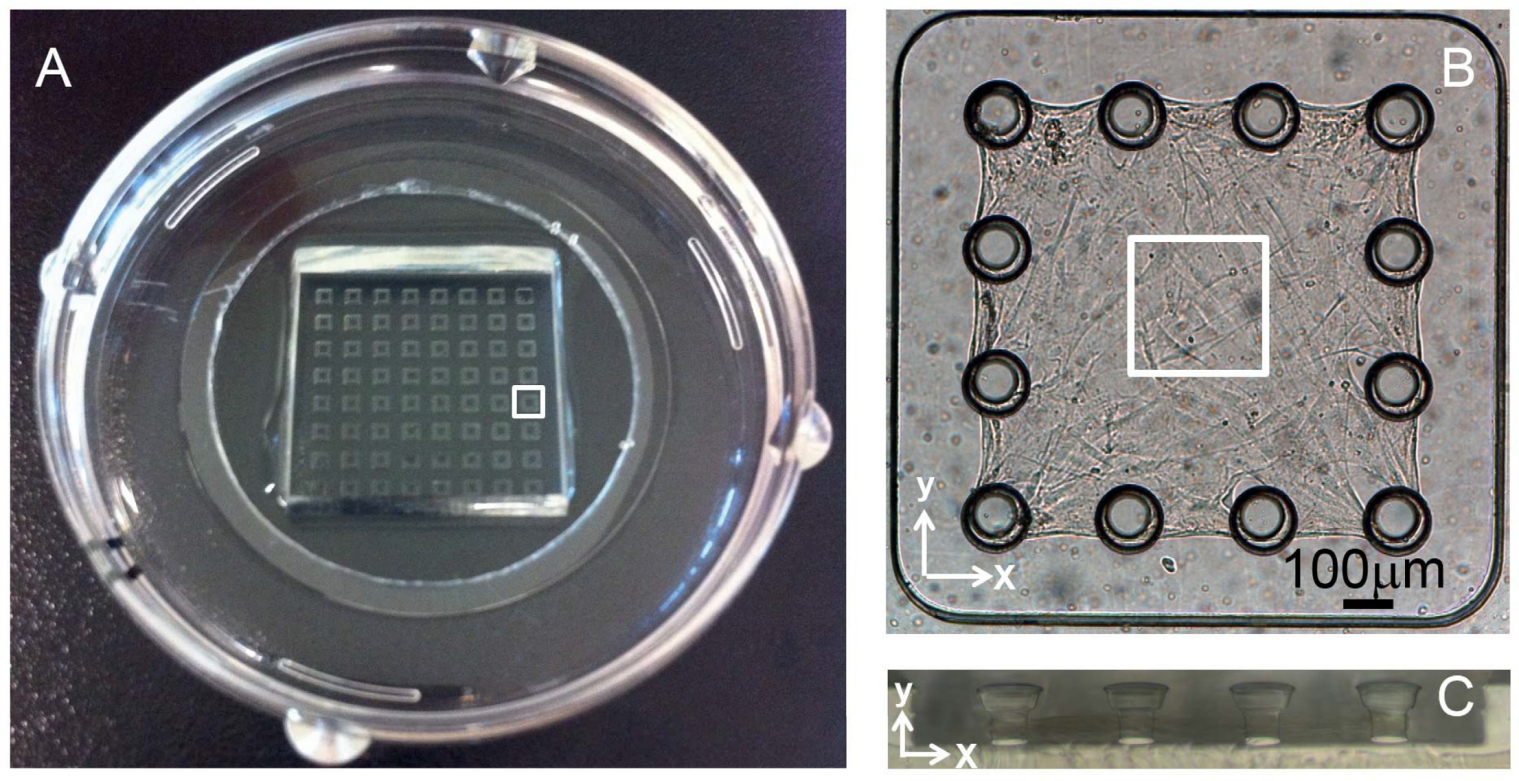
Model platform of tissue morphogenesis to generate fibroblast-contracted collagen microtissues. (A) An 8x8 microwell PDMS template was adhered to a petri dish containing a glass bottom. The white square indicates one single well in which a microtissue was engineered, as depicted in (B): Top view of a biaxially constrained microtissue in a single well of 1125×1125μm, composed of rat tail collagen type I mixed with MEFs. After contraction, tissues measure approximately 800×800×50μm. The white square indicates the area scanned with confocal microscopy (246×246μm) (C) Side view of 4 posts from one microwell. Post diameter was 75μm at the base and 125μm at the top, with a total height of 125μm.

**Fig 2:**
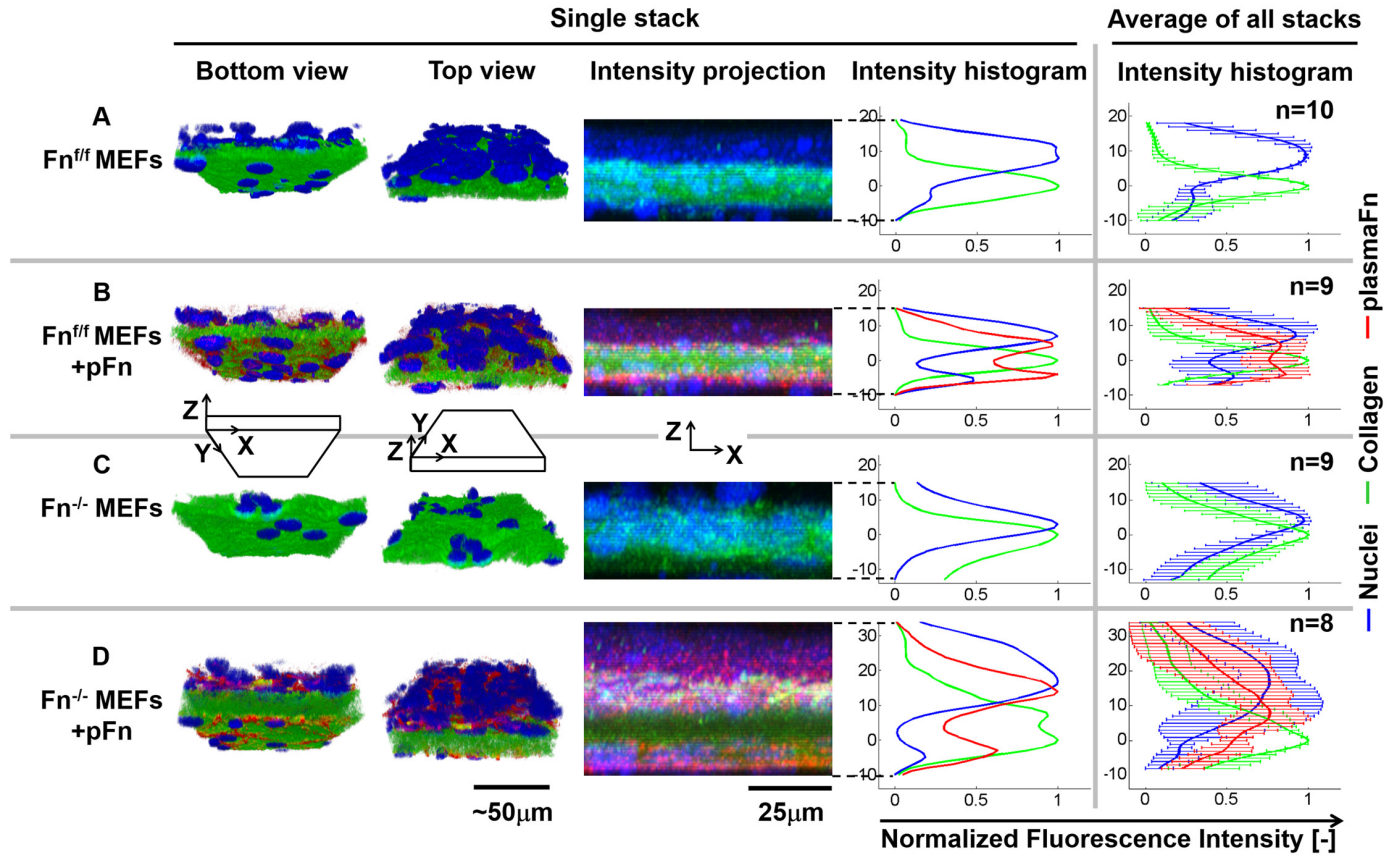
Fibronectin contributes to the depth-dependent separation of cells and collagen scaffold in microtissues. Microtissues, cultured for 72h, were either engineered from Fn^f/f^ MEFs in the absence (A) or presence of plasma fibronectin (B), or from Fn^-/-^ MEFs in the absence (C) or presence of plasma fibronectin (D). The depth-dependent distributions of cells and ECM components in a single tissue are represented by volume rendering of a cropped section of the Z-stack using Imaris software in a bottom and top view (1^st^ and 2^nd^ column) and a maximum projection cross-section of a cropped section of the tissue Z-stack with associated depth-dependent intensity histogram from the full Z-stack (3^rd^ and 4^th^ column, nuclei in blue; collagen in green and plasma fibronectin in red)). Tissue depth in the histograms is represented as the distance from the location in the tissue with maximum collagen intensity (0μm). The depth-dependent intensity histogram of all tissues combined is depicted in the 5^th^ column, which represents the raw data. Fn^f/f^ MEFs produce shell like tissues, where cells reside on both sides of the collagen core (A and B). However, in absence of fibronectin, Fn^-/-^ MEFs predominantly reside in the collagen core (C). Fn^-/-^ MEFs supplemented with fibronectin not only migrate towards tissue surfaces, but also assemble fibrillar fibronectin, primarily at the top surface (D). Percentage overlap of collagen and nuclei at the tissue bottom, core and top are quantified in S2 Fig.

To address the question of how fibronectin impacts cell-induced microtissue morphogenesis, Fn^-/-^ MEFs and Fn^f/f^ MEFs were mixed with collagen type I and seeded in microwells containing tissue-constraining posts (Fig 1A–1C). Fibronectin-depleted FBS-rich (10%) growth medium was added in all experiments with or without supplementation of exogenous plasma fibronectin. Microtissues produced by Fn^f/f^ MEFs developed into shell-like tissues (Fig 2A and 2B and S1A, S1 and S2 Movies). The core of the collagen scaffold was sparsely populated by cells whereas the periphery contained a high cell density (Figs 2B and S2B and S2D; S2 Movie). Contrary to the cells, plasma fibronectin was found to be more evenly distributed throughout the tissue (Figs 2B and S2B and S2D) as it can directly bind to collagen gel fibers independent of the presence of cells [10]. This confirms previous findings that 3T3 fibroblasts initially immersed in isotropic collagen gels drive the formation of a shell-like tissue morphology [10,15]. In contrast, microtissues contracted by Fn^-/-^ MEFs displayed a strikingly different depth-dependent distribution of the cells 72h after seeding. In the absence of fibronectin, the overall distribution of Fn^-/-^ MEFs in the collagen scaffold remained more homogenous (Figs 2C and S2B; S3 Movie), i.e. distributions of nuclei and collagen highly overlapped (S2B Fig). Adding exogenous plasma fibronectin at the start of the Fn^-/-^ MEFs culture, restored the shell-like composition of their floxed equivalents (Fig 2B), with plasma fibronectin being present throughout the complete tissue and also covering the collagen core (Figs 2D and S2B and S2E; S4 Movie). Interestingly, a much thicker fibrillar layer of plasma fibronectin was assembled by Fn^-/-^ MEFs at the tissue top surface, different from Fn^f/f^ MEFs equivalents, also visible from overall tissue thickness (Fig 2B vs 2D). The formation of shell-like tissues in the presence of plasma fibronectin for both cell types mostly established between 48 and 72h (S1A and S1B Fig; for floxed cells S5, S6 and S1 Movies (24h, 48h and 72h, respectively) and for knockout cells S7, S8 and S3 Movies (24h, 48h and 72h, respectively)).

### Exposure to fibronectin enhances Fn^-/-^ MEF migration speed

**Fig 3:**
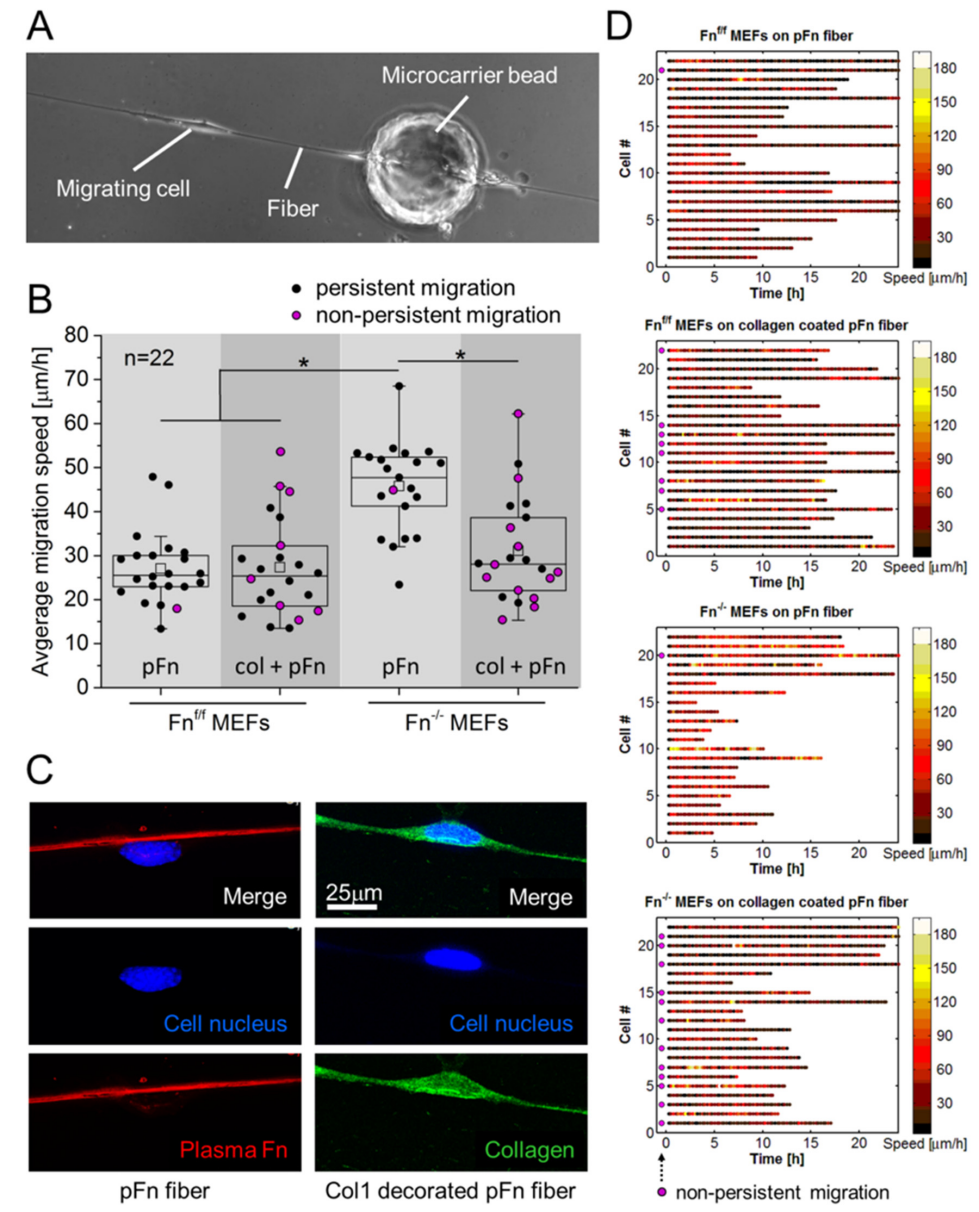
Average cell migration speed and persistence along fibers depends on the type of ECM coating. (A) Experimental setup for assessing migration speed and persistence of cells on manually pulled plasma fibronectin fibers. MEFs migrated from microcarrier beads onto fibronectin fibers, either left untreated, or decorated with rat tail collagen type I monomers. (B) Migration speed of single cells was tracked every 15min during 24h and averaged; speed was measured only for single cells (collectively migrating cells were excluded) using the fiber as their substrate. Non-persistently migration cells, i.e. cells that changed migration direction at least once, are marked by a magenta-colored circle. Fn^-/-^ MEFs, compared to Fn^f/f^ MEFs, displayed a significant increase in migration speed on pFn fibers (* p<0.05). Decorating pFn fibers with collagen however significantly reduced migration speed of Fn^-/-^ MEFs, to attain values that lacked a significant difference with Fn^f/f^ MEFs on uncoated fibers. Migration speed of Fn^f/f^ MEFs was not affected by the fiber coating. Migration persistence was negatively affected by collagen decoration, i.e. both Fn^-/-^ and Fn^f/f^ MEFs changed migration direction significantly more often, when compared to their counterparts migrating on pFn fibers. A significant difference in migration speed between non-persistent and persistent cells was not detected. (C) Confirmation of the presence of collagen coating after termination of the migration assay (at 24h). The stained collagen is unlikely to originate from the migrating Fn^-/-^ MEFs, since a specific rat-antibody for collagen is used, the medium does not contain ascorbic acid, and performed experiments were short-term (24h). (D) Raw data of cellular migration speed on manually pulled plasma fibronectin (pFn) fibers, untreated or decorated with collagen I monomers. For each condition, a number of 22 cells (y-axis) were tracked every 15min in at least 2 independent experiments. The color of each dot represents the migration speed (corresponding to the speed, indicated in the bar at the right) of a single cell at a specific time point (x-axis). Although manual tracking of the cells from one time point to the next is susceptible to the introduction of human errors by misinterpretation of the cell location, average cell speed as represented in panel B, is hardly affected. Cells were tracked for 24h maximum, however when encountering other cells, or when leaving the fibronectin fiber, cell tracking was terminated. Since Fn^-/-^ MEFs had a high migration speed, they more quickly encountered other cells, and were therefore often tracked for a shorter time. Non-persistent cells are indicated by a magenta-colored circle just next to the y-axis.

To further understand why Fn^-/-^ MEFs continued to reside within the collagen scaffold in the absence of fibronectin, we asked whether exposure to fibronectin versus collagen directly affects cell migration speed and persistence. Therefore, cell migration speed and persistence was quantified in single cell assays where fibroblast-populated beads were seeded on manually pulled plasma fibronectin fibers [32] (Fig 3A), since we know that the conformation of fibrillar fibronectin, which is often stretched by the cells, is far more extended compared to that of fibronectin adsorbed to flat glass substrates [48] or directly to collagen gels [10] and that the fibronectin conformational distribution within these single fibers closely resembled those observed in a native 3D extracellular environment [32]. The fibers were either left uncoated or were decorated with rat tail collagen type I (matching that of the 3D scaffolds in this study). The aim here was thus to construct fibers that are similar in their physical properties (diameter, rigidity, etc.), but present either fibrillar fibronectin versus collagen 1-decorated fibronectin fibers. To assess solely how cell migration is affected by the presence of different ECM components presented by single fibers, the migration speed and persistence of individual MEFs was tracked using fibronectin-depleted growth medium. Two major observations can be made: First, while the Fn^f/f^ MEFs and the Fn^-/-^ MEFs were highly persistent walkers on the single plasma fibronectin fibers, collagen I decoration increased the turn-around events for both (Fig 3B and 3D). Almost half of both Fn^f/f^ and Fn^-/-^ MEFs changed migration direction over the course of the observation (Fig 3B and 3C, magenta-colored circles). Second and perhaps surprising, the migration speed of Fn^-/-^ MEFs on plasma fibronectin fibers was significantly higher compared to Fn^f/f^ MEFs (Fig 3B and 3D), but decorating the plasma fibronectin fibers with collagen significantly reduced the migration speed of the Fn^-/-^ MEFs to levels approaching those of Fn^f/f^ MEFs (Fig 3B), while collagen coating had little effect on the migration speed of the Fn^f/f^ MEFs. However, persistently migrating cells on collagen decorated fibers did not show a significantly different average speed when compared to non-persistent cells (assessed for both Fn^f/f^ and Fn^-/-^ MEFs individually using Student’s T tests). The efficacy of the collagen coating covering the Fn-fibers was verified by staining for the different components after having tracked the cells in real-time (Fig 3C).

### Fibronectin enhances the generation of cell traction forces

**Fig 4:**
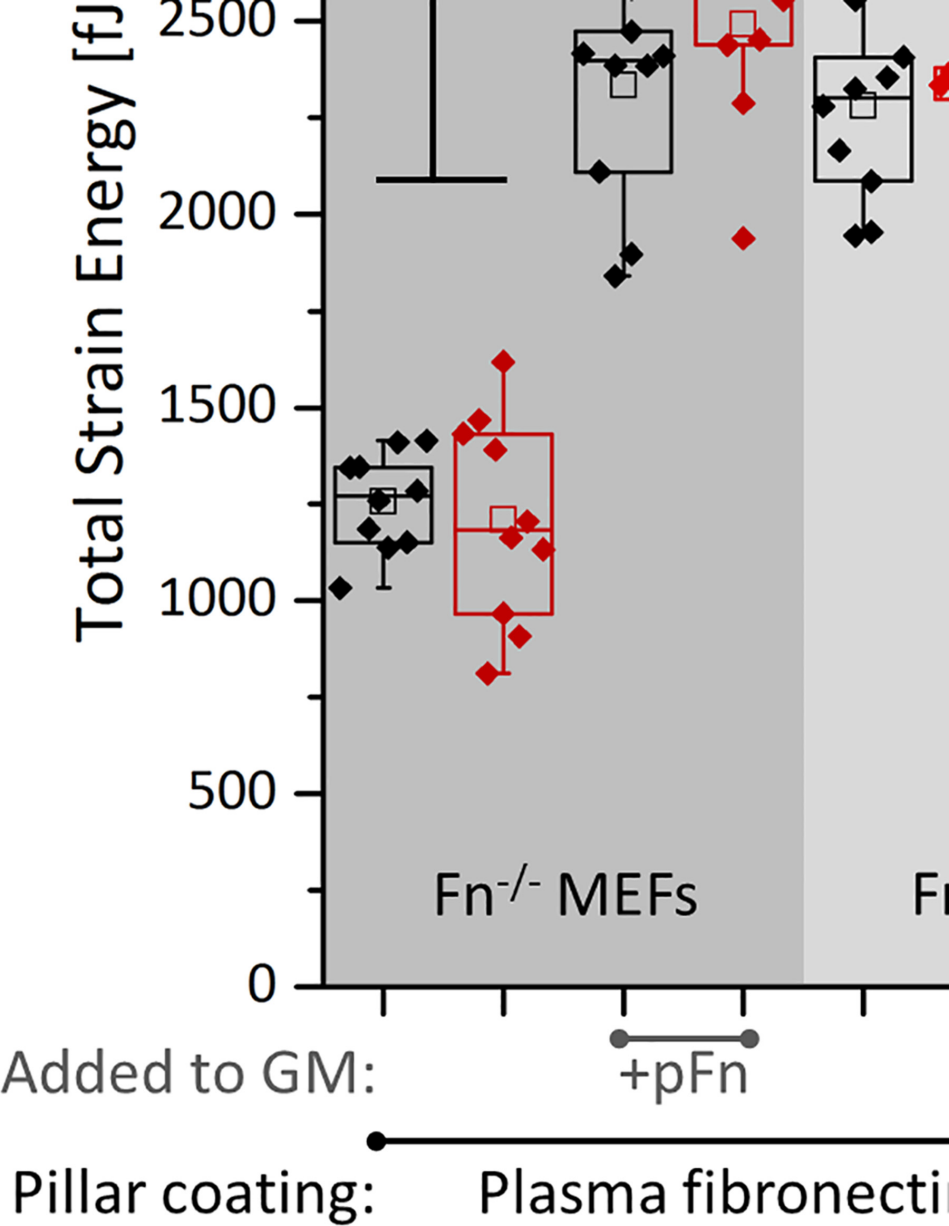
Total strain energy of fibroblasts on SU-8 nanopillar arrays. (A) Experimental setup for measuring cell-induced nanopillar displacement: Pre-labeled MEFs (red) were seeded on plasma fibronectin (pFn) or vitronectin-coated nanopillars for 30min in Fn-depleted FBS-rich (10%) media (SEM image added at the right, showing how fibroblasts deflect the posts). Pillars have a diameter of 250nm, a height of 1.5μm and center to center distance was 800nm. Medium was supplemented without or with 45nM fibronectin (full-length, or the 40k, 70k or 120kDa fragments), during cell seeding, and pillar displacements were measured 2 and 24 hours after cell seeding. (B) Total strain energy per cell, 2h and 24h after seeding, for different pillar coatings (fibronectin versus vitronectin) and in the presence of exogenous pFn and of its fragments. For pillars coated either with fibronectin or vitronectin, pFn in the medium upregulates total strain energy generated by Fn^-/-^ MEFs approaching those of Fn^f/f^ MEFs. On pFn-coated nanopillars, addition of pFn in the medium significantly increased total strain energy per Fn^-/-^ MEF attaining values that equal Fn^f/f^ MEFs. Vitronectin coating significantly decreased the strain energy by Fn^-/-^ MEFs (indicated by #). On vitronectin coated pillars, the 70kDa fragment significantly increased strain energy of Fn^-/-^ MEFs (indicated by £), which is likely to be caused by a possible contamination of full length fibronectin in this particular fragment, S4 Fig. However, only full length exogenously added pFn rescues total strain energy by Fn^-/-^ MEFs on fibronectin or vitronectin-coated pillars to meet their floxed (Fn^f/f^ MEFs) counterparts. For a representation of the average strain energy per pillar, see S3 Fig.

Since cell-generated forces drive tissue morphogenesis [2,3,49,50,51,52], we next asked how the presence of plasma fibronectin in the medium might affect the generation of cell traction forces. Fn^f/f^ or Fn^-/-^ MEFs were seeded on nanopillar substrates coated with fibronectin, or vitronectin (which binds αv, but not α5 integrins), with or without exogenous plasma fibronectin (full-length or fragments) in the medium (Fig 4A). Total strain energy [53,54] was calculated from the pillar deflections measured 2h and 24h after cell seeding (Fig 4B; S3 Fig for average strain energy per pillar). A slight increase was observed for the total strain energy between 2h and 24h for almost all groups, associated with an increased spreading area (as visible from the decreasing average strain energy in time per pillar, S3 Fig), in agreement with previous literature [55]. On fibronectin-coated nanopillars, Fn^-/-^ MEFs generated significantly lower (~45%) strain energy in the absence of exogenously added fibronectin to the growth medium for both time points when compared to Fn^f/f^ MEFs. The addition of exogenous plasma fibronectin to the medium fully rescued total strain energy by Fn^-/-^ MEFs to match their floxed equivalents. This is surprising since in both cases, cells are exposed to plasma fibronectin present as coating on the nanopillars.

To detect the extent to which fibronectin-coating alone affected the total strain energy, the experiment was repeated for Fn^-/-^ MEFs on vitronectin coated nanopillars, again for both 2h and 24h after seeding. Vitronectin coating significantly reduced total strain energy (~35%), compared to fibronectin coating (Fig 4B; S3 Fig for average strain energy per pillar). On vitronectin-coated pillars, the addition of exogenous plasma fibronectin to the medium again fully rescued the strain energy generated by Fn^-/-^ MEFs (Fig 4B; S3 Fig for average strain energy per pillar). Hence, fibronectin, both as substrate coating and to a far greater extend when present in the growth medium, increased the total cellular strain energy. Since fibronectin contains binding sites for various integrins [16], we next asked whether exogenously added full-length plasma fibronectin was required to upregulate cell traction forces, or whether certain fibronectin fragments, none of which can undergo fibrillogenesis, are equally efficient. Fn^-/-^ MEFs were therefore seeded on vitronectin coated nanopillars and exposed to fibronectin fragments supplemented to the medium. The 70kDa N-terminal fibronectin fragment (repeats FnI_1-9_ and FnII_1-2_) [56] provides integrin binding sites for α5β1 and contains the collagen/gelatin binding region [16]. The 40kDa C-terminal fibronectin fragment (repeats FnIII_12-14_) [56] contains the heparin binding domain II, while the 120kDa central fragment (repeats FnIII_1-11_) [56] provides many integrin binding sites, among others the RGD epitope, the α5β1 epitope and the synergy site [16,41]. Although addition of the 70kDa fibronectin fragment significantly increased the total cellular strain energy (~50%) at both time points, it did not reach the strain energy levels generated in the presence of full-length fibronectin (Fig 4B). This 70kDa-mediated increase is likely to be caused by a modest contamination of what could be full length fibronectin, as detected by Western blotting of the 70k fragment using a polyclonal fibronectin antibody (S4 Fig). Also the other fibronectin fragments used (40kDa and 120kDa) did not increase total strain energy, irrespective of the time point. Thus, the presence of full-length fibronectin in the medium, and not of its fragments, is required to fully rescue the cell traction forces of Fn^-/-^ MEFs.

### Full-length fibronectin is required for the development of steep cell density gradients

**Fig 5:**
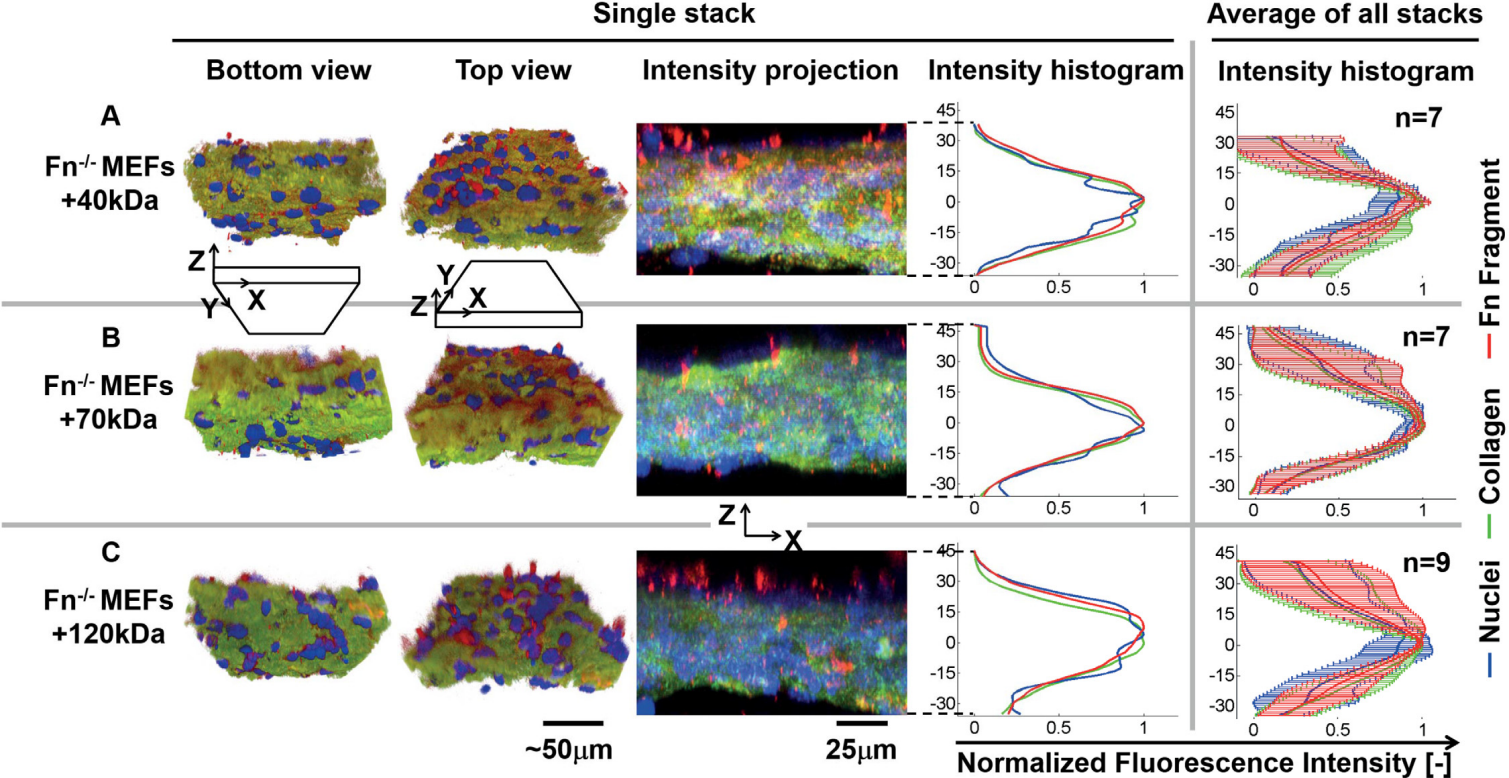
Fibronectin fragments (40, 70, 120kDa) do not contribute to the depth-dependent separation of cells from the collagen core. Microtissues, cultured for 72h, were engineered from Fn^-/-^ MEFs mixed with rat tail collagen type I in the presence of fibronectin fragments, either 40kDa (A), 70kDa (B) or 120kDa (C). In the presence of the fibronectin fragments, Fn^-/-^ MEFs predominantly resided in the collagen core. The depth-dependent distributions of cells and ECM components in a single tissue are represented by volume rendering using Imaris software in a bottom and top view (1^st^ and 2^nd^ column) and a maximum projection cross-section of the tissue with the associated depth-dependent intensity histogram (3^rd^ and 4^th^ column, nuclei in blue; collagen in green and fibronectin fragments in red, all stained using a polyclonal antibody). Tissue depth in the histograms is represented as the distance from the location in the tissue with maximum collagen intensity (0μm). The depth-dependent intensity histogram of all tissues combined is depicted in the 5^th^ column, which represents the raw data. Percentage overlap of collagen and nuclei at the tissue bottom, core and top are quantified in S2 Fig.

Since the magnitude of traction forces and developing tissue heterogeneity may be linked, Fn^-/-^ MEFs were mixed with collagen I to produce microtissues, while being exposed to the different fibronectin fragments (40, 70 or 120kDa) that were supplemented to the growth medium from the start of culture. Even though all of these fragments were found to bind to the collagen gel, cell nuclei remained homogeneously distributed throughout the collagen gel (Figs 5 and S2C; S9–S11 Movies). Tissue contraction appeared to be further limited by the addition of the fragments, since the tissues were generally observed to be thicker than Fn^-/-^ MEFs in collagen only (Fig 2C). Thus, cells require full-length fibronectin for tissue morphogenesis, i.e. the accumulation of fibroblasts at the surface of the contracted collagen gels.

### Gradient of fibronectin conformations, from the core to the microtissue periphery

**Fig 6:**
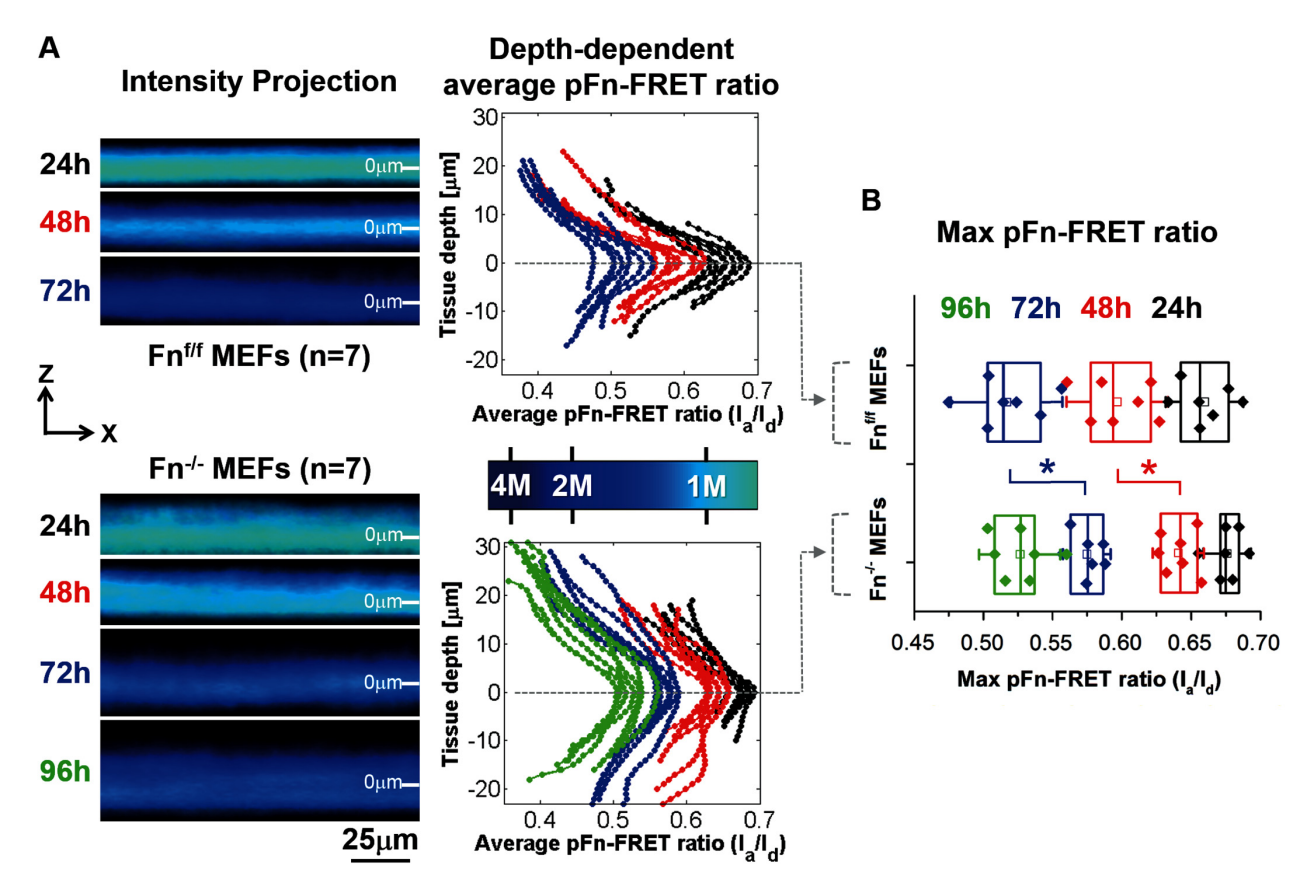
The Fn-FRET probe reveals a steep gradient of fibronectin conformations. (A) MEFs embedded in collagen type I gels were incubated up to 96h in Fn-containing growth medium supplemented with trace amounts of FRET-Fn. The left panel displays representative cropped average intensity projection images showing the depth-dependent FRET signal in microtissue cross sections at the indicated time points. The right panel shows the distribution of the FRET ratio throughout all tissue Z-stacks for each measured tissue (images were taken every 1μm in the Z-direction). The maximum FRET ratio was used to set tissue depth to 0μm. The colored bar in between both graphs represents the color-coded pFn-FRET ratio, corresponding to the left panel. Values correspond to FRET ratios for fibronectin in solution progressively denatured by GdnHCl, as depicted in S5 Fig. For both the left and right panel, the average pFn-FRET-ratios decreased from the tissue core towards the outer surfaces. In addition, although the shape of the fibronectin conformational distribution remains unchanged with increasing culture time, average FRET ratios drop with time, whereby Fn^-/-^ MEFs lag behind on Fn^f/f^ MEFs approximately 24h (see also panel B). (B) Comparison of maximum pFn-FRET values between groups (representing the average value at 0μm tissue depth), confirming that maximum values for Fn^-/-^ MEFs at time point 48h and 72h are significantly higher compared to Fn^f/f^ MEFs. The * represents a significant difference (p<0.05) between time-matching groups, assessed with student t-test.

Since a previous study showed that FRET-labeled plasma fibronectin is assembled by 3T3 cells into fibrillar ECM at the microtissue periphery, much more than in the core where it was mostly observed to adsorb to collagen fibers [10], FRET-labeled plasma fibronectin was added to the growth medium of Fn^-/-^ versus Fn^f/f^ MEFs-populated microtissues to assess its depth-dependent conformational gradients. The FRET signal (calibrated using previously designed methods [31], S5 Fig) was detected after 24, 48, 72 and for Fn^-/-^ MEFs also 96h after the start of culturing. Fibronectin was observed to be highly stretched at the tissue surfaces (corresponding to low FRET) that gradually decreased (corresponding to high FRET) towards the core (Figs 6A, S12–S18 hin for Fn^-/-^ MEFs, tissues grew thicker over time (distributions widened in time, similar to the results shown in S1B Fig). For both Fn^-/-^ and Fn^f/f^ MEFs, the complete FRET histograms of fibronectin moved over time to lower FRET values, while roughly maintaining their shape (Fig 6A, graphs at the right). This decrease in average Fn-FRET with culture time happens as the cells contract the collagen gels, coinciding with a possible change in the ratio of collagen-absorbed, more compact fibronectin vs extended fibrillar fibronectin assembled from the growth medium by fibroblasts [10]. Remarkably, from the 48h time point onwards, the FRET-ratios in the core of the collagen gels contracted by the Fn^-/-^ MEFs lagged behind in time approximately 24h, compared to Fn^f/f^ MEFs.

## Discussion

Since the underpinning mechanisms that drive the formation of cell gradients and gradients of ECM composition in tissues and engineered tissue scaffolds are not yet resolved [9,10,11,12,20,21,22,27,28,41,42,43,45,57], we exploited 3D collagen-derived microtissues as model system to investigate the role of fibronectin in such processes. During contraction of the collagen gels by fibroblasts, cells accumulate at the microtissue surface (S1 and S2 Figs), in agreement with previous observations [10,13]. Using fibronectin knockout (Fn^-/-^) versus floxed mouse fibroblasts (Fn^f/f^), we found that fibronectin plays a crucial role in directing the accumulation of cells at the tissue surface (Fig 2). The current study revealed that the absence of fibronectin abolished the accumulation of cells at the tissue surfaces (Fig 2C). The microtissue platforms allowed us to show that the presence of exogenous plasma fibronectin, but not of the 40kDa, 70kDa or 120kDa fibronectin fragments, rescued the ability of knockout fibroblasts to accumulate at the tissue surface (Figs 2 and 5). This is especially interesting in the context of the ability for full-length fibronectin to undergo fibrillogenesis, which is lacking for the fibronectin fragments alone [41,58,59]. This observation supports the possibility that gradients in the conformation of fibronectin, as probed here by our Fn-FRET sensor, perhaps in combination with changing fibronectin-to collagen ratios, direct tissue morphogenesis by guiding cells towards the tissue surfaces.

Cell migration assays on single fibers revealed that Fn^-/-^ MEFs migrate faster on plasma fibronectin fibers, when compared to collagen-decorated fibronectin fibers (Fig 3). In contrast, Fn^f/f^ MEFs show similar migration speeds, irrespective of the Fn-fiber coating. The positive effect of fibronectin on the migration properties of Fn^-/-^ MEFs was previously shown in a scratch assay, where the addition of plasma or recombinant wild type fibronectin increased wound closure by Fn^-/-^ MEFs on 2D collagen coated surfaces [42,59]. Also, C2C12 cells migrate further on fibronectin-coated surfaces, compared to gelatin [60]. In 3D, fibronectin depletion from plasma clots prevented fibroblast invasion [27], and invasion of collagen constructs by tumor cells is enhanced upon addition of exogenous fibronectin [61].Here we show that collagen decoration of fibronectin fibers enhanced non-persistent migration by both Fn^f/f^ and Fn^-/-^ MEFs (Fig 2). This may explain why Fn^-/-^ do not accumulate at tissue surfaces in the absence of fibronectin. Although Fn^f/f^ MEFs were equally affected by collagen decoration of fibronectin fibers in decreasing persistent migration, our data show that their ability to produce cellular fibronectin allows them to accumulate at microtissue surfaces, even in the absence of plasma fibronectin. A possible explanation lies in the key difference between the manually pulled fibers and 3D: as the Fn^f/f^ cells migrate on collagen-decorated fibers, they produce and assemble cellular fibronectin matrix underneath themselves which might override the collagen “effect” seen in the accelerated movement of the Fn^-/-^ cells. In 3D, the cells can encounter endogenous fibronectin produced by other cells while they migrate through the ECM. While we acknowledge that directly relating observations made from cells migrating on manually pulled fibers to phenomena in 3D cell-assembled ECM is difficult, manually pulled fibers and cell-derived ECM fibers do share similarities. The conformational distribution of Fn is similar on both types of fibers [32]. Another constraint that potentially limits Fn^-/-^ MEFs to accumulate at tissue surfaces on collagen scaffolds is the required presence of MMPs for migration through collagen ECM of small pore sizes [62]. Since fibronectin is known to upregulate the production of various MMPs [26], this could be a means by which Fn^-/-^ MEFs travel through the collagenous rich ECM to accumulate at the tissue surface. Additionally, fibronectin was shown to induce cell proliferation of Fn^-/-^ MEFs in 3D collagen constructs [43,63]. The addition of fibronectin to Fn^-/-^ MEFs in our setup very likely also resulted in increased proliferation, as visible from the volume rendered images, compare Fig 2C with 2D. However, since the tissue core is completely deprived of cells with the addition of fibronectin after 72h, it seems unlikely that local proliferation at tissue surfaces has been the sole factor to drive the special separation of cells and ECM, i.e. cell migration through the ECM is proposed to have played a dominant role. Taken together with our finding that Fn^-/-^ MEFs embedded in a 3D collagen scaffold resided in the collagen core (Fig 2), and the above-mentioned literature, we conclude that the presence of fibronectin is essential and drives the formation of a microtissue with well-defined gradients in cell density and ECM composition.

We furthermore observed that plasma fibronectin added to the medium upregulated the generation of traction forces by Fn^-/-^ MEFs pulling on nanopillars coated with plasma fibronectin compared to vitronectin as probed 2h and 24h after cell seeding (Fig 4). In agreement with these results, previous work showed that type I collagen gel contraction by Fn^-/-^ MEFs is limited when fibronectin fragments or vitronectin was added to the gel, when compared to adding full-length fibronectin [41]. Additionally, fibronectin upregulates 3T3 fibroblast contractility and migration compared to RGD-only modified substrata [64], and that vitronectin and fibronectin, although they share binding sites for several integrins, namely αvβ1 [65], αIIbβ3 (platelet integrin), α8β1 and αvβ3 [44], are distinct in αvβ5 (vitronectin) versus α5β1 and αvβ6 integrins (fibronectin) [44]. Our finding that fibroblasts apply enhanced traction forces, giving rise to an enhanced total strain energy to fibronectin versus vitronectin coated pillars is in agreement with the literature, i.e. integrin α5β1 but not αvβ3 was shown to increase the ability of fibroblasts to sustain force [66], and quantitative proteomics linked αv-class integrins to a GEF-H1-RhoA pathway coupled to the formin mDia1, but not myosin II, and α5β1 integrins to a RhoA-Rock-myosin II pathway [67]. Strikingly, only the presence of full-length plasma fibronectin in the medium, and not of its fragments, could recover the forces by which Fn^-/-^ MEFs pulled on either vitronectin or fibronectin-coated nanopillars. The 70kDa fragment partially rescued the forces; however this is very likely the result of a minor contamination with possible full length fibronectin as shown by Western blot (S4 Fig). Consequently, not just the integrin junctions formed with surface-bound vitronectin, but the presence of fibronectin in the medium positively contributes to cellular traction force generation, suggesting activation of additional integrins through adsorption of fibronectin from solution to the cell membrane surface. This mode of integrin activation is supported by previous studies performed on fibroblasts adhering to RGD-functionalized supported lipid membranes and showing that integrin activation occurs before the cells apply tensile forces to those integrins [68]. Such effect, i.e. the effect of a soluble protein in the growth medium on cell behavior was also recently demonstrated for FAK-activation in freely suspended cells in growth medium by adding soluble collagen [39]. Although a direct extrapolation of results from nanopillars to 3D constructs is difficult to make, lower force generation in the presence of fibronectin fragments, compared to full-length fibronectin, corroborates with our observation in the 3D constructs that tissue compaction in the Z-direction appeared to be limited in the presence of fibronectin fragments, compared to all other groups. The latter agrees with a study by Hocking et al. [41], who also observed limited gel contraction in the presence of the same fragments.

The high cell densities at the microtissue surfaces correlated with fibronectin that showed significantly lower FRET than regions in the core of the microtissue (Fig 6). Since we show that the fibronectin FRET-profile changes significantly at the length scale of a single cell, a depth-dependent gradient of fibronectin conformations in these microtissues exists, from more compact in the core of the contracted collagen to more extended and partially stretched fibrillar fibronectin at the tissue periphery. Since it was shown recently [39] that fibronectin-mediated FAK activation is dependent on the mechanical tension acting on fibronectin, which may expose its otherwise hidden synergy site to integrin α5, the accumulation of fibroblasts at the microtissue surfaces may thus originate from increasing the fraction of collagen-adsorbed to stretched fibrillar fibronectin conformations, from the tissue core to the surface, which might thereby provide conformational-dependent guidance cues. Different fibronectin conformations could also indirectly act as a cell guiding cue by differential exposure of binding sites for chemoattractant growth factors (e.g. PDGF, VEGF, TGFβ and FGF) [19,69,70,71]. The preferred accumulation of cells at the top surface of microtissues, compared to the bottom, is a feature that has been observed before as well [10], but the causative factors for this phenomenon are still unknown. Microtissues have previously been observed to contract around posts within 12h [15,72] and are thus suspended up to 75μm above the substrate at a stage well before asymmetries in cellular distributions from apical to basal appear. Although oxygen limitations were shown to only be a limiting factor in tissues that exceed 200μm [14], and our microtissues are approximately 4 times thinner (~50μm), accessibility of larger molecules by cells at the basal side may be limited especially in the first phase of gel contraction when compared to the apical side. We thus acknowledge that the difference in cell density from apical to basal may be driven by chemotaxis and thereby presents an artifact of the model system. The central part of microtissues contains high collagen densities (Fig 2) and high FRET-values (Fig 6), while moving towards the tissue surface, collagen density and FRET progressively decrease. The fibronectin FRET-ratios in microtissues populated by Fn^-/-^ MEFs were shifted to significantly higher values at the time points 24h, 48h and 72h (Fig 6B), respectively, indicating that Fn^-/-^ MEFs may contract the tissues at a slower rate. Locations in the tissue that contain high collagen densities may correlate with a high degree of fibronectin that is absorbed to the collagen, as shown earlier for collagen-adsorbed fibronectin compared to fibrillar fibronectin [10]. The FRET decrease over time in the core may indicate that the initially adsorbed fibronectin may be gradually released from the collagen over time, thus changing the ratio between absorbed and fibrillar fibronectin.

In conclusion, fibroblasts accumulated at microtissue surfaces in the presence of full-length fibronectin. Building order from randomly assembled cells requires cell migration and force generation, and both were shown here to be promoted by full-length fibronectin on manually assembled fibers and nanopillars, respectively. Unraveling fibronectin-induced mechanisms that drive spatial organization of cells and ECM, and thus tissue morphogenesis, is of vital interest in development, as well as to build and maintain engineered tissue functionality and durability.

## Materials and Methods

### Microtissue fabrication

A previously developed model system was used [73,74], composed of an array of 8 by 8 microwells, with each well containing 12 posts with a narrow base and wide top (total height 125μm) in a square setup (Fig 1). Before use, the model systems were made hydrophilic by plasma treatment (1 min at 100W) to facilitate gel seeding. Subsequently, they were sterilized by treatment with 70% alcohol for 30min and UV exposure for 15min, and finally treatment with 0.2% Pluronic F127 (BASF) for 2 min to reduce cell adhesion.

Fibronectin-knockout (Fn^-/-^) or fibronectin floxed (Fn^f/f^) mouse embryonic fibroblasts (MEFs) [25,47,75] were obtained from Professor Rainhard Faessler (Max-Planck Institute for Biochemistry, Martinsried, Germany). Cells were added to a gel mixture of growth medium (DMEM low glucose with glutamax, Gibco; 10% Fn-depleted FBS, Biowest; 1% penicillin/streptomycin, Gibco), collagen type I (final concentration 1mg/mL, rat tail, BD Bioscience) and sodium hydroxide to neutralize the pH of the acidic collagen. Gels were mixed with cells to attain a final density of 10^6^ MEFs per mL gel. All MEFs used were below passage 10. To each microwell, approximately 0.16μL of the cell-gel mixture was pipetted (the volume of a single well). During this procedure, microwells were kept on ice to prevent evaporation of the gel. Gels subsequently polymerized in an incubator at 37°C, 5% CO_2_ for 10min. To prevent dehydration of the gel during polymerization, the petri dish was inverted and sterile water was added to the lid. After polymerization, growth medium was added to the gels, which contracted around the posts in approximately 6h [73,74]. Where indicated, the growth medium was supplemented with 40nM fibronectin (unlabeled plasma fibronectin, single or double (FRET) labeled plasma fibronectin, or fibronectin fragments, i.e. 40kDa, 70kDa or 120kDa). Supplemented concentrations of full-length fibronectin, as indicated in this manuscript, are derived from the molecular weight of the monomer and thus theoretically contain twice the amount of binding sites compared to added fragments. Fibronectin fragments are purchased from Sigma and obtained through proteolysis of human plasma fibronectin. FRET-labeled fibronectin was always mixed with unlabeled fibronectin at a ratio of 1:10 to avoid signal from intermolecular FRET. Fibronectin was added just after tissue seeding and remained in the culture throughout the complete experiment. Engineered microtissues were cultured up to 96h.

### Visualization and distribution analysis

After the designated culture period, microtissues were fixed in 10% formalin for 30min and permeabilized for 30min in 0.5% Triton-X in PBS (visualization of FRET-fibronectin was performed in absence of triton-X treatment). The following antibodies were used for specific staining of ECM components and cell nuclei: 1F10C2 (Chondrex) for rat tail collagen type I, ab23750 (Abcam) for fibronectin, and Dapi (Fluka) for cell nuclei. Exogenously added plasma fibronectin was pre-labeled with Alexa488 (A20000, Invitrogen). Z-stack fluorescent images were taken with a confocal microscope (Leica SPX5). The pinhole of the photo-multiplier was set to the optimal width for the used 63x, 1.2N.A. water immersion lens (Zeiss Plan Apo CS). Photo-multipliers accepted wavelength regions of 418–461nm for Dapi, 498-544nm for fibronectin (alexa488), or 643-739nm for collagen (alexa633). Microtissues anchored to the posts were visualized through the glass bottom of the petri dish. Individual images were taken every 1μm through the complete tissue depth, which measured 246 x 246μm each (as shown in Fig 1B). No additional image processing was performed. A representation of the distribution of cells, collagen and fibronectin throughout the tissue depth was obtained by processing maximum projection images from the Z-stacks using ImageJ, which were cropped in the x-direction to 75μm for a clear representation of the cell and protein distributions in the Z-direction. Emission intensity profiles throughout the tissue depth were obtained using ImageJ for each tissue, using the complete stack, not the cropped version. For each condition, intensity profiles were normalized, i.e. maximum emission for each channel was set to 1, after which profiles within the same condition were averaged. Averaging was performed after shifting the intensity profiles over the x-axis to have the maximum collagen intensity slice at the exact same location (set at a tissue depth of Z equals 0μm), explaining the lack of a standard deviation at this specific depth for collagen. Tissue depth is represented as distance from the location in the tissue with maximum collagen intensity, which therefore equals the arbitrary value of 0μm.

Imaris software was used to produce volume rendered representative parts (cropped to 100x100μm) of the 3D stacks from single tissues. Contrast and brightness of individual channels (nuclei, collagen and fibronectin) were visually optimized for each tissue. Direct comparison between samples in terms of intensity is not valid. The volume rendered images merely aim to give the reader a 3D view of the differences between cellular distributions throughout tissue depth.

The distribution of cells and ECM (collagen and fibronectin) throughout microtissue thickness was quantified as follows. For each tissue, the tissue core was defined by all Z-stack slices from the collagen channel that had a normalized fluorescent intensity of 0.5 or higher (S2A Fig, vertical dashed line intersecting with the green line: representing collagen), thereby defining 3 regions in the tissue, i.e. bottom, core and top. Intensity values belonging to the three different regions, were summed and normalized to the total sum of intensity values, thus normalizing to 100%. Since high intensity values for the collagen channel are taken as a reference, the tissue core contains the majority of the collagen. Tissue bottom and top of the microtissues thus have limited amount of collagen and represent the basal and apical side, respectively. Subsequently, the sum of intensity values for the Dapi and fibronectin channels were calculated in the same way, but always based on the ‘bottom, core, top’-distribution of the collagen channel. The visual representation of these distributions thus represent the fraction of cells or fibronectin that overlap with the collagen core, and the top and bottom surface of the microtissues. To determine significant differences between bottom, core and top, One-Way-ANOVA, Bonferroni post hoc test was applied to the Dapi and fibronectin distributions.

### Fibronectin isolation, fluorescent labeling, and FRET imaging and analysis

Fibronectin was isolated, (FRET-)labeled and analyzed, including a characterization via chemical denaturation curves, as previously described [31]. In short, fibronectin was purified from human plasma (Swiss Red Cross). The cysteines on the fibronectin modules FnIII_7_ and FnIII_15_ were labeled with Alexa Fluor 546 as acceptor fluorophores (A, maleimide, Molecular Probes), whereas amines were randomly labeled with Alexa Fluor 488 as donor fluorophores (D, succinimidyl ester, Molecular Probes). The labeling ratio of Fn-DA was determined by measuring the absorbance of at 280, 498 and 556nm. On average, 13.5 donors and 4.0 acceptors were labeled on each fibronectin dimer. Upon stretching fibronectin, the distance between the multiple acceptors and donors increases resulting in a reduction in the acceptor to donor intensity ratios, here referred to as FRET (I_A_/I_D_). The use of multiple donors and acceptors prevents the calculation of FRET efficiency, but is sensitive to a large range of conformations. FRET–labeled fibronectin in PBS was stored at -20°C and added to cultures immediately after thawing.

All images that involve FRET measurements were acquired with an Olympus FV-1000 confocal microscope with a 40×0.9 N.A. water immersion objective, with a set pinhole diameter of 200μm. Slices from each Z-stack were acquired at 318x318μm with a slice spacing of 1μm. All images were acquired with a 3xKalman line averaging. Tissues were excited with a 488nm laser, while emission of the donor and acceptor were detected using photomultiplier tubes (PMTs) with detection ranges set at 514–526nm (donor channel) and 566–578nm (acceptor channel). All images were acquired with the exact same settings, i.e. PMT voltage, laser power and pixel dwell time, to prevent hardware related misinterpretation of the FRET-values. Donor bleed into the acceptor channel was determined by imaging the emission of 488-labeled Fn in both channels. Donor bleed was constant throughout tissue depth and averaged 0.207 ± 0.001, meaning that approximately 21% of the donor channel was present in the acceptor channel. Because of the linear relation, i.e. donor bleed being constant throughout tissue depth, the shape of the FRET-curves were not affected. Obtained Z-stack images were analyzed using a modified version (automation of FRET measurements for Z-stacks was implemented) of a previously designed script in Matlab [10,31]. FRET-distributions were obtained by calculating the average FRET value for each image in the Z-stack throughout the complete tissue depth. Previous work [10] showed that the FRET-signal itself is not affected by imaging through collagen-based microtissues and thus truly represents a conformation change in the molecule.

The batch calibration of fibronectin FRET-ratios was performed using the technique as previously described [31] by dissolving FRET-labeled fibronectin at different concentrations of the denaturant GdnHCl. The settings for visualization of the calibration curve using the Olympus microscope were maintained when imaging FRET in 3D constructs.

### Migration assay

Migration speed and persistence of MEFs was assessed on manually pulled plasma fibronectin fibers, based on a previously developed system [32,76]. Fibronectin fibers were pulled from solution (0.4 mg/mL plasma fibronectin in PBS) and deposited on silicone sheets (SMI .005” NRV G/G 40D, Specialty Manufacturing, Saginaw, MI) that were cut to fit a 4-well LabTek chamber (#155382, Thermo Scientific). Fibronectin fibers were washed gently with PBS and treated with 4% BSA in PBS for 60min. After washing with PBS, fibers were left untreated or were decorated for 1h with rat tail collagen I (10μg/mL in DMEM at 37°C, BD Bioscience). Subsequently, all fiber-containing silicone sheets were treated overnight with 20 mg/mL BSA, 10% PenStrep in PBS. After the migration assay, fibers were imaged for the presence of collagen decoration for validation purposes.

Cytodex microcarrier beads (C3275, Sigma) coated with MEFs to near confluence were used as sources of cells that could migrate away onto the fibronectin fibers. Briefly, MEFs (0.3million) were suspended in 1mL growth medium and added to untreated 24-well plates (351147, Falcon). Subsequently, 200μL of Cytodex beads (10^4^ beads per mL of PBS) were added to the wells. MEFs attached to the beads in the subsequent 72h of culturing. Cell-populated beads were transferred to fibronectin fibers (uncoated or coated with collagen) in fibronectin-depleted FBS-rich (10%) growth medium. After 6h, MEFs started to migrate from the beads onto the fibers. Migration was imaged time-lapsed using an Axiovert 200 M inverted microscope (Carl Zeiss). Images were taken every 15min for 24h at multiple locations per condition.

Migration speed was quantified using ImageJ software by selecting the position of individual cells at every time point. Only those MEFs were tracked that migrated as a single cell and used the fiber as guidance. Non-persistent cells were marked when changing direction at least once during tracking.

### Nanopillar experiments

SU-8 nanopillar arrays (SU-8 2000.5, Microchem) were fabricated by a combination of nanosphere lithography and plasma etching, attached to glass coverslips, as previously described [77]. Nanopillars were coated with vitronectin (10nM) or plasma fibronectin (10nM) via incubation for 30min. MEFs, pre-labeled with membrane dye DiI (Invitrogen), were allowed to spread on coated nanopillars at 7,500cells/cm^2^ in fibronectin-depleted growth medium. Growth medium was, where indicated, supplemented with plasma fibronectin or the fibronectin fragments; 40kDa, 70kDa and 120kDa (final concentration 45nM). After 2h or 24h, cells were fixed in 10% formalin for 30min after which pillar deflection and cell morphology was imaged using a Leica confocal microscope SP5 with a 63x, oil immersion, 1.4 N.A. objective. Pillar displacements were measured using Diatrack 3.03 (Powerful Particle Tracking, Semasopht). Atomic force microscopy was used to determine the spring constant of single nanopillars, resulting in an average value of 79 ± 3 nN/μm. To calculate the total strain energy for each cell, the displacement of each post was squared and multiplied by half the spring constant of a nanopillar and summed for all pillars that connected to that cell, as described previously [53,54].

### Statistics

For the migration assay, One-Way ANOVA was used to determine the effect of the coating and its interaction with migration speed. For cellular force measurements on the nanopillars, One-Way ANOVA was used to determine the effect of the combination of cell type and growth medium content (using the same coating, either fibronectin or vitronectin) and its interaction with total strain energy. For comparing the distributions of nuclei and fibronectin relative to the collagen core (S2 Fig), One-Way ANOVA was used to detect significant differences in the percentage of cells or Fn between bottom, core and top of tissues from different conditions. For ANOVA, all p-values were corrected with the Bonferroni criterion. Student’s t-tests were performed to check for significant differences between MEFs (analysis performed for Fn^f/f^ and Fn^-/-^, separately) that showed persistent migration vs non persistent migration on collagen decorated pFn fibers. Student T-tests were also performed to test for significant differences between maximum fibronectin FRET-value and cell type (Fn^-/-^ versus Fn^f/f^ MEFs) for each time point (24h, 48h and 72h) and for total strain energy of Fn^-/-^ MEFs on fibronectin vs vitronectin coated nanopillars. P-values <0.05 were considered statistically significant. The indicated sample size originates from at least 2 different wells, in which differences between the independent experiments were not observed.

## Supporting Information

S1 Fig The presence of fibronectin contributes to microtissue morphogenesis in a time-dependent manner. Tissues either contained Fn^f/f^ MEFs (A) or Fn^-/-^ MEFs (B) in rat tail collagen gels, supplemented with plasma fibronectin in the growth medium. MEFs produced shell like tissues from 48-72h, where at 72h cells reside primarily apically from the collagen core, with sparse cells at the basal side of the collagen core. The depth-dependent distributions of cells and ECM components in a single tissue are represented by volume rendering using Imaris software in a bottom and top view (1^st^ and 2^nd^ column) and a maximum projection cross-section of the tissue with associated depth-dependent intensity histogram (3^rd^ and 4^th^ column, nuclei in blue; collagen in green and plasma fibronectin in red)). Tissue depth in the histograms is represented as the distance from the location in the tissue with maximum collagen intensity (0μm). The depth-dependent intensity histogram of all tissues combined is depicted in the 5^th^ column, which represents the raw data. Strikingly, Fn^-/-^ MEFs assemble more fibronectin at the tissue surface, visible at 72h, compared to their floxed counterparts. To note, the data presented for Fn^f/f^ MEFs at 72h resemble Fig 2A, while data for Fn^-/-^ MEFs at 72h resemble Fig 2C. Percentage overlap of collagen and nuclei at the tissue bottom, core and top are quantified in S2 Fig.
(TIF)

S2 Fig Quantification of the depth-dependent collagen-nuclei overlap in microtissues. (A) Method of quantifying the distributions of cells and ECM (collagen and fibronectin) throughout microtissue depth, as explained in the materials and methods section. The collagen core is defined as the sum intensities from an arbitrary threshold of 0.5, resulting in three regions, i.e. bottom, core and top. Subsequently, percentages based on sum intensity for the three different tissue sections are calculated for nuclei (blue curve), collagen (green curve) and fibronectin (red curve). (B-D) Distributions of nuclei, collagen and fibronectin in the different tissue zones (bottom, core and top), in which error bars represent standard deviations. Analyses represent histograms presented in Fig 2 (S2B Fig), Fig 5 (S2C Fig: fragments) and S1 Fig A (S2D Fig: time-course of Fn^f/f^ MEFs) and S1B Fig (S2E Fig: time-course Fn^-/-^ MEFs). Analysis of statistical differences between the percentages in ‘bottom’, ‘core’ and ‘top’ are performed using One-Way-ANOVA with a Bonferroni post hoc test.
(EPS)

S3 Fig Cellular traction forces (2 and 24h after seeding), represented by average strain energy per pillar, for different pillar coatings (fibronectin versus vitronectin) and in the presence of exogenously added pFn and of its fragments. Compared to total strain energy (Fig 4), similar trends are visible, however values at 24h for average strain energy are slightly lower, resulting from an increased spreading area between 2h and 24h after cell seeding.
(EPS)

S4 Fig Western blot analysis of the 70k fibronectin fragment shows a possible contamination with full length fibronectin. 1μg of the 70k fibronectin fragment was loaded and probed with a rabbit polyclonal antibody against fibronectin (ab23750, Abcam). Although the majority of the protein consists of the 70k fragment, a possible contamination of fibronectin monomer can be seen at band size 250. This may explain the slightly elevated cellular traction forces measured using the nanopillar assay.
(EPS)

S5 Fig Calibration of fibronectin FRET-ratios in solution upon progressive denaturation. FRET-labeled fibronectin was dissolved in different concentrations of the denaturant GndHCl. The loss of secondary structure of the fibronectin protein starts beyond the concentration of 1M GndHCl [31,48] (corresponding to acceptor-versus donor intensities, Ia/Id = 0.63 and higher). The protein is completely denatured at GndHCl concentrations of 4M (la/ld = 0.37). Left: Probability density distributions of FRET-fibronectin in solutions at different GndHCl concentrations. Right: The denaturation curve containing average values from 3 individual measurements of the probability density distributions are presented in combination with the standard deviation.
(EPS)

S1 Movie Representative Z-Stack of a tissue from Fig 2A: Fn^f/f^ MEFs in a collagen gel at 72h of culturing. (AVI)

S2 Movie Representative Z-Stack of a tissue from Fig 2B: Fn^f/f^ MEFs in a collagen gel at 72h of culturing with exogenously added plasma fibronectin. (AVI)

S3 Movie Representative Z-Stack of a tissue from Fig 2C: Fn^-/-^ MEFs in a collagen gel at 72h of culturing. (AVI)

S4 Movie Representative Z-Stack of a tissue from Fig 2D: Fn^-/-^ MEFs in a collagen gel at 72h of culturing with exogenously added plasma fibronectin. (AVI)

S5 Movie Representative Z-Stack of a tissue from S1 Fig A 24h: Fn^f/f^ MEFs in a collagen gel at 24h of culturing with exogenously added plasma fibronectin. (AVI)

S6 Movie Representative Z-Stack of a tissue from S1A Fig 48h: Fn^f/f^ MEFs in a collagen gel at 48h of culturing with exogenously added plasma fibronectin. (AVI)

S7 Movie Representative Z-Stack of a tissue from S1B Fig 24h: Fn^-/-^ MEFs in a collagen gel at 24h of culturing with exogenously added plasma fibronectin. (AVI)

S8 Movie Representative Z-Stack of a tissue from S1B Fig 48h: Fn^-/-^ MEFs in a collagen gel at 48h of culturing with exogenously added plasma fibronectin. (AVI)

S9 Movie Representative Z-Stack of a tissue from Fig 5A: Fn^-/-^ MEFs in a collagen gel at 72h of culturing supplemented with the 40kDa fibronectin fragment. (AVI)

S10 Movie Representative Z-Stack of a tissue from Fig 5B: Fn^-/-^ MEFs in a collagen gel at 72h of culturing supplemented with the 70kDa fibronectin fragment. (AVI)

S11 Movie Representative Z-Stack of a tissue from Fig 5C: Fn^-/-^ MEFs in a collagen gel at 72h of culturing supplemented with the 120kDa fibronectin fragment. (AVI)

S12 Movie Representative FRET-distribution in a tissue from Fig 6A 24h: Fn^f/f^ MEFs in a collagen gel at 24h of culturing supplemented with FRET-labeled fibronectin. (AVI)

S13 Movie Representative FRET-distribution in a tissue from Fig 6A 48h: Fn^f/f^ MEFs in a collagen gel at 48h of culturing supplemented with FRET-labeled fibronectin. (AVI)

S14 Movie Representative FRET-distribution in a tissue from Fig 6A 72h: Fn^f/f^ MEFs in a collagen gel at 72h of culturing supplemented with FRET-labeled fibronectin. (AVI)

S15 Movie Representative FRET-distribution in a tissue from Fig 6B 24h: Fn^-/-^ MEFs in a collagen gel at 24h of culturing supplemented with FRET-labeled fibronectin. (AVI)

S16 Movie Representative FRET-distribution in a tissue from Fig 6B 48h: Fn^-/-^ MEFs in a collagen gel at 48h of culturing supplemented with FRET-labeled fibronectin. (AVI)

S17 Movie Representative FRET-distribution in a tissue from Fig 6B 72h: Fn^-/-^ MEFs in a collagen gel at 72h of culturing supplemented with FRET-labeled fibronectin. (AVI)

S18 Movie Representative FRET-distribution in a tissue from Fig 6B 96h: Fn^-/-^ MEFs in a collagen gel at 96h of culturing supplemented with FRET-labeled fibronectin. (AVI)
