# Correction: Full-Length Fibronectin Drives Fibroblast Accumulation at the Surface of Collagen Microtissues during Cell-Induced Tissue Morphogenesis

**DOI:** 10.1371/journal.pone.0165354

**Published:** 2016-10-19

**Authors:** The PLOS ONE Staff

Fig 4, “Total strain energy of fibroblasts on SU-8 nanopillar arrays,” is only partially visible. Please view Fig 4 here. The publisher apologizes for the error.

**Fig 4:**
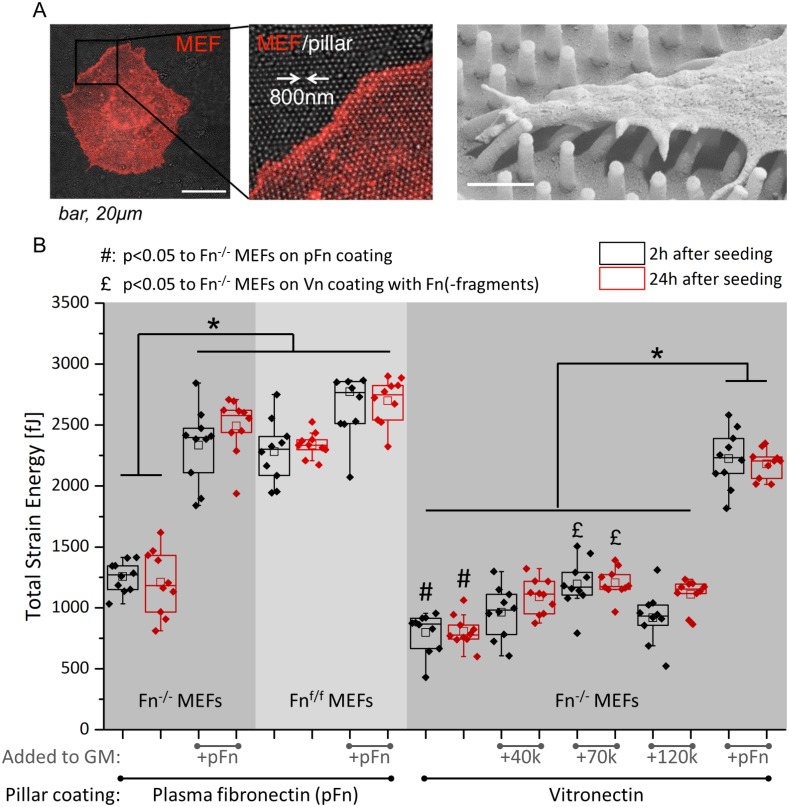
Total strain energy of fibroblasts on SU-8 nanopillar arrays. (A) Experimental setup for measuring cell-induced nanopillar displacement: Pre-labeled MEFs (red) were seeded on plasma fibronectin (pFn) or vitronectin-coated nanopillars for 30min in Fn-depleted FBS-rich (10%) media (SEM image added at the right, showing how fibroblasts deflect the posts). Pillars have a diameter of 250nm, a height of 1.5μm and center to center distance was 800nm. Medium was supplemented without or with 45nM fibronectin (full-length, or the 40k, 70k or 120kDa fragments), during cell seeding, and pillar displacements were measured 2 and 24 hours after cell seeding. (B) Total strain energy per cell, 2h and 24h after seeding, for different pillar coatings (fibronectin versus vitronectin) and in the presence of exogenous pFn and of its fragments. For pillars coated either with fibronectin or vitronectin, pFn in the medium upregulates total strain energy generated by Fn^-/-^ MEFs approaching those of Fn^f/f^ MEFs. On pFn-coated nanopillars, addition of pFn in the medium significantly increased total strain energy per Fn^-/-^ MEF attaining values that equal Fn^f/f^ MEFs. Vitronectin coating significantly decreased the strain energy by Fn^-/-^ MEFs (indicated by #). On vitronectin coated pillars, the 70kDa fragment significantly increased strain energy of Fn^-/-^ MEFs (indicated by £), which is likely to be caused by a possible contamination of full length fibronectin in this particular fragment, S4 Fig. However, only full length exogenously added pFn rescues total strain energy by Fn^-/-^ MEFs on fibronectin or vitronectin-coated pillars to meet their floxed (Fn^f/f^ MEFs) counterparts. For a representation of the average strain energy per pillar, see S3 Fig.
