# Supplementary figures and images for: Full-Length Fibronectin Drives Fibroblast Accumulation at the Surface of Collagen Microtissues during Cell-Induced Tissue Morphogenesis
Source: PLoS One. 2016 Aug 26;11(8):e0160369. doi: 10.1371/journal.pone.0160369 (PMC5001707; doi:10.1371/journal.pone.0160369)

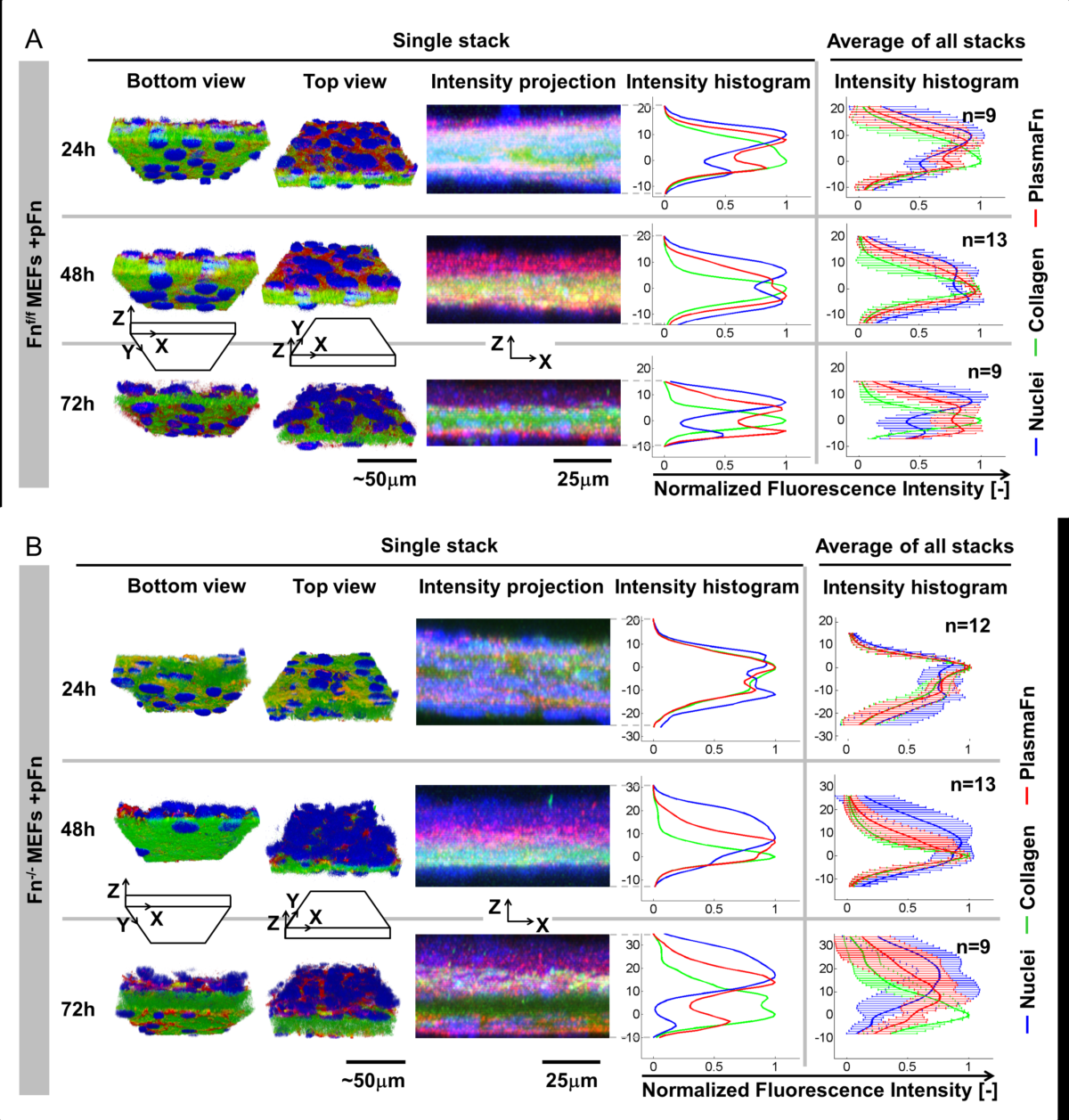

Supplement: S1 Fig — Tissues either contained Fnf/f MEFs (A) or Fn-/- MEFs (B) in rat tail collagen gels, supplemented with plasma fibronectin in the growth medium. MEFs produced shell like tissues from 48-72h, where at 72h cells reside primarily apically from the collagen core, with sparse cells at the basal side of the collagen core. The depth-dependent distributions of cells and ECM components in a single tissue are represented by volume rendering using Imaris software in a bottom and top view (1st and 2nd column) and a maximum projection cross-section of the tissue with associated depth-dependent intensity histogram (3rd and 4th column, nuclei in blue; collagen in green and plasma fibronectin in red)). Tissue depth in the histograms is represented as the distance from the location in the tissue with maximum collagen intensity (0μm). The depth-dependent intensity histogram of all tissues combined is depicted in the 5th column, which represents the raw data. Strikingly, Fn-/- MEFs assemble more fibronectin at the tissue surface, visible at 72h, compared to their floxed counterparts. To note, the data presented for Fnf/f MEFs at 72h resemble Fig 2A, while data for Fn-/- MEFs at 72h resemble Fig 2C. Percentage overlap of collagen and nuclei at the tissue bottom, core and top are quantified in S2 Fig. (TIF) [file pone.0160369.s001.tif]
